# Regulation of Dendritic Cell Immune Function and Metabolism by Cellular Nutrient Sensor Mammalian Target of Rapamycin (mTOR)

**DOI:** 10.3389/fimmu.2018.03145

**Published:** 2019-01-14

**Authors:** Julia P. Snyder, Eyal Amiel

**Affiliations:** ^1^Predoctoral student of the Cellular, Molecular, and Biomedical (CMB) Sciences Graduate Program at the University of Vermont, Burlington, VT, United States; ^2^Department of Biomedical and Health Sciences, College of Nursing and Health Sciences, University of Vermont, Burlington, VT, United States

**Keywords:** dendritic cell (DC), mTOR, immune metabolism, glycolysis, metabolism regulation

## Abstract

Dendritic cell (DC) activation is characterized by an acute increase in glucose metabolic flux that is required to fuel the high anabolic rates associated with DC activation. Inhibition of glycolysis significantly attenuates most aspects of DC immune effector function including antigen presentation, inflammatory cytokine production, and T cell stimulatory capacity. The cellular nutrient sensor mammalian/mechanistic Target of Rapamycin (mTOR) is an important upstream regulator of glycolytic metabolism and plays a central role in coordinating DC metabolic changes and immune responses. Because mTOR signaling can be activated by a variety of immunological stimuli, including signaling through the Toll-like Receptor (TLR) family of receptors, mTOR is involved in orchestrating many aspects of the DC metabolic response to microbial stimuli. It has become increasingly clear that mTOR's role in promoting or attenuating inflammatory processes in DCs is highly context-dependent and varies according to specific cellular subsets and the immunological conditions being studied. This review will address key aspects of the complex role of mTOR in regulating DC metabolism and effector function.

## Introduction

As the quintessential professional antigen presenting cells of the immune system, dendritic cells (DCs) play a central role in coordinating both innate and adaptive immune responses through efficient recognition and uptake of extracellular material and the potent ability to provide both presented antigen and costimulatory signals required for proper T lymphocyte activation (1). DC activation is typically initiated by Pattern Recognition Receptor (PRR) interactions with microbe-associated ligands, as has been exhaustively characterized for the Toll-like receptor (TLR) family of innate immune receptors (2–4). Signaling downstream of these receptors induces important transcription and translation programs in DCs that are essential for the induction of their immune effector function. While DCs share many of the innate immune features of other myeloid cells of the mononuclear phagocyte lineage such as the expression of PRRs, efficient endocytic, and phagocytic clearance of extracellular matter, and robust induction of cytokine-driven inflammatory response upon activation, DCs undergo a distinct cellular program of maturation that is affiliated with their important contributions to T cell activation. These latter functions include the processing and presentation of antigens on MHC molecules, the upregulation of co-stimulatory molecule expression, and the induction of chemokine receptor expression that drives DC migration to secondary lymphoid organs where DCs encounter and activate T lymphocytes through cognate antigen interactions (1, 5, 6).

While historically the field of immunology has focused on immune cell regulation at the transcriptional and translational levels, the recent emergence of the field of “immunometabolism” has provided the scientific community with a new framework for thinking about immune cell activation; specifically, how nutrient availability and usage controls the cellular effector functions important for immunological protection. One of the cornerstone findings of recent advances in the field is the observation that immune cell activation, in both the lymphoid and myeloid lineages, is broadly characterized by an increased flux of glucose metabolism, often termed in the literature as “aerobic glycolysis” as both a historical nod to the analogous “Warburg metabolism” described in cancer cells [reviewed in Potter et al. (7)] and to emphasize that the increase in glucose metabolism is not systemically induced by hypoxic conditions (8–18). Analogous to lymphocyte dependence on glucose metabolism for activation [reviewed in (17, 19)], TLR stimulation of DCs induces significant upregulation of aerobic glycolysis that is required for the survival and immune effector function of both human and mouse DCs (10–12, 15, 20, 21). As a cellular nutrient sensor and important upstream regulator of glycolytic metabolism, Mammalian Target of Rapamycin (mTOR) plays a central role in coordinating DC metabolic changes and immune responses. mTOR's role in cell biology is far-reaching and highly complex, regulating a diverse network of cellular responses including cell metabolism, energy homeostasis, protein translation, cellular differentiation, and proliferation, autophagy, and cell survival. Despite this complexity, the existence of highly selective, non-toxic, and FDA-approved mTOR inhibitors such as rapamycin has allowed the research community to broadly interrogate the role of mTOR function in DC biology at both the cellular and organism/patient level. As the role of mTOR in immune cell development and autophagy regulation have been covered comprehensively by previous reviews [reviewed in (22–24)], these aspects of mTOR biology will not be covered in depth. Instead, the focus of this review will be to highlight and discuss the current understanding of mTOR-dependent metabolic regulation of DC function.

## The Role of mTOR in Cellular Metabolism

The hierarchical regulation of cellular metabolism and energy homeostasis can be functionally partitioned into two opposing “programs,” anabolism and catabolism, each governed by a distinct central upstream regulator. Energetic anabolism, generically characterized by reduced metabolic activity coupled to energy conservation and production, is controlled by AMP-activated protein kinase (AMPK) in response to low cellular ATP levels or nutrient starvation [reviewed in (25)]. Catabolism, contrastingly comprised by high rates of energy expenditure for nutrient breakdown and molecular biosynthesis, is controlled by mTOR complex activity [reviewed in (26)]. Not surprisingly, these two processes cross-regulate each other, most notably by AMPK inhibition of mTOR activation. While AMPK has been implicated in important aspects of DC biology (10, 27, 28), it is a notably understudied aspect of DC metabolic biology and this review will focus primarily on mTOR-mediated metabolic regulation of DCs.

The mTOR protein itself functions as a required component of two major signaling complexes, mTOR complex 1 (mTORC1) and mTOR complex 2 (mTORC2). While mTORC1 is primarily responsible for cellular energy expenditure and protein translation, mTORC2 serves an important role as a positive regulator of mTORC1. For the purposes of this review, “mTOR activity” will refer to mTORC1 functions unless otherwise noted. It is notable that while mTOR promotes sustained catabolism of carbohydrates, it concurrently supports the *de novo* synthesis of lipids, proteins, and amino acids, serving as an important checkpoint in converting increased cellular fuel consumption into processes such as cell division and protein production that have obvious implications for broad physiological responses, including those carried out by immune cells (29). As a downstream target of the PI3K/Akt signaling axis, mTOR activation in DCs can be initiated by a number of immunologically relevant factors, including cytokine signaling, growth factor signaling, and PRR signaling. In light of this, mTOR is positioned as a critical molecule integrating immunological stimuli into changes in cellular metabolism that regulate protein translation events required for the immunological function of these cells. The role of mTOR in governing immune cell homeostasis and the use of mTOR inhibitors as viable immunoregulatory strategies continue to be of intense interest to the field (23).

## DC Commitment to Aerobic Glycolysis

Activation of DCs via TLRs promotes significant upregulation of aerobic glycolysis, which regulates the immune function of both human and mouse DCs (10–12, 18, 20, 21, 30, 31). To date, ligands for both MyD88 -dependent and -independent TLR members have been shown to result in an acute upregulation of glycolysis (20), as well as ligands for the C-type Lectin Receptors Dectin-1/2 (21), suggesting that this metabolic reprogramming may be a broadly conserved feature of PRR signaling. A wide variety of approaches, including inhibition of glycolysis through culture with 2-deoxy-glucose (2DG), pharmacological inhibition of glycolysis-regulating signaling pathways, and genetic silencing of rate-limiting glycolysis enzymes, have demonstrated that loss of glycolytic capability significantly impairs DC effector functions, including antigen presentation, co-stimulatory molecule expression, chemotaxis, cytokine secretion, and T lymphocyte stimulatory capacity (10–12, 18, 20, 21, 30, 31). The prevailing consensus has emerged that acute, and in some cases sustained, metabolic commitment to elevated rates of glucose catabolism are an essential metabolic requirement for proper DC activation. We have previously argued that DC metabolic reprogramming can be functionally partitioned into two temporal phases governed by distinct signaling events (32): (1) an acute induction of glycolysis occurring within minutes of TLR activation that supports the high biosynthetic demand associated with early DC maturation for several hours (20); (2) a long-term commitment to glycolysis in subsets of nitric oxide (NO) -producing DCs that is required for their metabolic adaptation to NO-mediated mitochondrial toxicity (12, 15).

### Acute Glycolytic Reprogramming in DCs Is mTOR-Independent

Rapid induction of glycolysis in DCs, occurring within minutes of TLR stimulation, is controlled by a PI3K/TBK1/IKKε/Akt signaling axis that promotes the rapid translocation of Hexokinase 2 (HK2) to the mitochondria which supports the rapid flux of glucose catabolism associated with DC maturation (20). Glucose is rapidly consumed by activated DCs and glucose-derived carbons are primarily invested in pentose phosphate pathway (PPP) metabolism, lactate production, and citrate synthesis via the mitochondrial citrate shuttle, the latter presumably supporting fatty acid synthesis associated with endoplasmic reticulum, and Golgi body -dependent translation and secretory pathways that control inflammatory cytokine production (16, 20). The source of glucose that fuels this early activation comes from both the import of extracellular glucose, and the catabolism of intracellular glycogen pools that these cells possess in the resting state (18). The acute induction of glycolysis mediated by the PI3K/TBK1/IKKε/Akt signaling axis, conserved in multiple DC subsets in both mouse and human systems (20, 21, 31), is transient (lasting approximately 6–8 h) after which glycolysis levels gradually wane close to their pre-activation levels (20). The inability of mTOR inhibitors to attenuate this early wave of glycolysis indicates that mTOR activation is positioned downstream of early glycolysis commitment in DCs (12, 15, 20).

### Sustained Glycolytic Reprogramming in DCs Is mTOR-Dependent

While a number of studies have concluded that that mTOR, and one of its downstream transcription factors HIF1α, are required for DC glycolytic reprogramming (9, 30, 33–35), we and others have shown that this is primarily the case for the long-term commitment to glycolysis observed in NO-producing DCs that express inducible nitric oxide synthase (iNOS) and is independent of the acute glycolytic reprogramming events described above (12, 15). In iNOS-expressing DCs, largely restricted to inflammatory monocyte-derived DCs in the mouse and minor subsets of human DCs [previously reviewed in (32)], mTOR–dependent HIF1α activity promotes iNOS expression in TLR-activated DCs (30, 36). iNOS protein expression becomes detectable just as the acute induction of glycolysis begins to wane (12, 15), followed by NO-mediated suppression of DC mitochondrial activity (12, 15) through reversible inhibition of mitochondrial cytochrome c oxidase function (37, 38). Through its regulation of iNOS expression, mTOR regulates the long-term commitment of these cells to glycolytic metabolism in a NO-dependent manner (12, 15). Notably, mTOR inhibition decreases NO production and restores mitochondrial function in iNOS-expressing DCs which leads to increased metabolic flexibility and enhanced inflammatory activity in these cells (11). While the multifaceted and highly complex NO-independent impacts of mTOR on DC function are discussed in more detail below, it is impossible to ignore the important role of mTOR in regulating DC iNOS expression and the implications of this on DC metabolism.

### DC Lipid Metabolism

Metabolite tracing studies have shown that the rapid catabolism of glucose in TLR-stimulated DCs is closely linked with a number of biosynthetic pathways including the preferential generation of citrate through the TCA cycle (16, 20). Citrate production is understood to support fatty acid synthesis required for the expansion of endoplasmic reticulum and Golgi body cellular structures associated with DC activation (16, 20, 39, 40). While mTOR signaling is known to promote lipid biosynthesis (29), the explicit role of mTOR in regulating the citrate and fatty acid biosynthesis in stimulated DCs remains poorly defined. Nevertheless, the regulation of lipid metabolism in DCs has clear immunological relevance as there are notable instances where parasite infection of DCs leads to significant changes in lipid metabolism (41). In addition, LPS stimulation leads to specific increases in cellular ceramide concentration in DCs and immunogenic DCs and tolerogenic DCs display unique intracellular lipid profiles (42). With respect to cholesterol lipid metabolism, it is clear that this too is a highly important process in DCs. Liver X receptor, an important regulator of cholesterol metabolism, is implicated in promoting both DC differentiation and immune activation (43). Cholesterol hydroxylase activity is specifically upregulated by Type-I interferon signaling in macrophages and DCs (44), and both PRR and MHC molecules are associated with cholesterol-enriched lipid raft microdomains in the plasma membrane of DCs (45, 46). While it is logical that mTOR activity is involved in regulating these processes based on its role in other cell types, further work delineating mTOR's role in DC lipid metabolism is an important area for future investigation.

## mTOR Regulation of DC Effector Function

Because pharmacological inhibitors of mTOR function attenuate lymphocyte proliferation, mTOR signaling has classically been considered to play a broadly pro-inflammatory role in the immune system and has been used extensively for its systemic tolerogenic properties in the clinic. Recent studies at the cellular level have revealed that mTOR can exert both inflammatory and anti-inflammatory effects depending on the physiological context and cellular subsets in question. A summary of these findings for mTOR's documented impact on various aspects of DC effector function are delineated below.

### DC Maturation and Co-stimulatory Molecule Expression

While some studies have concluded that mTOR inhibition has minimal or contrasting effects on aspects of DC activation (47–49), other studies have argued that mTOR inhibition dramatically influences DC maturation in either a positive or negative direction. Many studies in both the mouse and human system have shown a negative impact on DC maturation (47, 50–52), while others have shown that mTOR inhibition can actually augment DC activation (10, 11, 15, 50). In one study, treatment with 1,25-dihydroxyvitamin D_3_ was shown to induce an mTOR-dependent tolerogenic phenotype in monocyte-derived human DCs (moDCs) that was characterized by decreased surface expression of CD80, HLA-DR, and CD86, and increased production of IL-10 (53). In mouse bone marrow -derived DCs (BMDCs), shRNA knocking down AMPK, a negative regulator of mTOR activity, increased costimulatory molecule expression while AMPK agonists decreased DC maturation (10). These studies show a rapid de-phosphorylation of AMPK upon LPS stimulation and that IL-10 attenuates LPS-mediated AMPK de-phosphorylation (10). Taken together, these findings are consistent with a model whereby reduced AMPK activity and concomitant mTOR induction serve as a “master switch” to promote DC maturation and immune function. Consistent with this, we and others have shown that CD40 and CD86 expression on mouse BMDCs and subsets of human DCs can be enhanced by mTOR inhibition during TLR stimulation of these cells (11, 15, 50). One of the most informative studies for resolving the published discrepancies on the role of mTOR in DC maturation, published by Haidinger et al. showed that mTOR inhibitors negatively regulate IL-4/GM-CSF -differentiated moDC activation, but augment maturation of freshly isolated myeloid DCs from human peripheral blood (50). In accordance with this, multiple studies have reported that the requirement for mTOR signaling in DC development and function varies with respect to the DC subsets in question (50, 54, 55). This intriguing idea, that mTOR exhibits disparate roles in unique DC subsets is further supported by the finding that different DC subsets engage distinct metabolic signatures to support their specialized function (56, 57). To this point, tolerogenic DCs are reported to exhibit an increased dependence on mitochondrial metabolism, in contrast to the glycolysis-centric phenotype observed for many inflammatory DC subsets (56, 57). The fact that mTOR activator is typically considered an upstream promoter of protein translation, it is interesting that mTOR inhibitors can actually augment proinflammatory molecule production in certain DC subsets (11, 15, 50). This phenomenon is not restricted to the role of mTOR in regulating iNOS expression as it has also been reported for circulating human DCs that do not produce NO upon activation (11, 50).

### Cytokine Production

The production of cytokines has been the most widely studied DC effector function with respect to the role of mTOR in these cells. Freshly isolated mouse splenic pDCs exhibit an mTOR -activated phenotype, and rapamycin-treated mouse and human pDCs show impaired secretion of multiple cytokines including Type I interferons, TNF-alpha, and IL-6 (58). In *L. monocytogenes* infected mice, rapamycin treatment protected animals from lethal challenge, and led to increased serum concentrations of IL-12p70, IFN-γ, and IL-6 (59). Treatment of human moDCs with the phytochemical cytopiloyne, which is reported to preferentially target mTORC2 signaling, lowers LPS-driven costimulatory molecules expression and inflammatory cytokine production (60). Another study identified a beta-Catenin/mTOR signaling axis as a primary driver of DC IL-10 production responsible for CD8+ T lymphocyte activation (61). Multiple studies have shown that inhibition of mTOR leads to decreased IL-10 production, often concomitant with increased production of inflammatory cytokines such as IL-12 and IL-6 (50, 62). Consistent with this, mTORC1 signaling by intestinal DCs has been shown to be required for IL-10 production and tolerance homeostasis in the gut (62). The connection between mTOR signaling and DC IL-10 production is particularly noteworthy because IL-10 has been shown to antagonize long-term glycolysis commitment in BMDCs (10). Upon LPS stimulation of whole blood from kidney transplant patients treated with rapamycin, IL-12p40, IL-6, TNF-α, and IL-1β levels were increased while IL-10 levels were decreased, compared to patient controls (63). Furthermore, rapamycin treatment of human CD14+ monocytes was shown to enhance IL-12p40, IL-12p70, and TNF-α production, and decrease IL-10 production, upon LPS stimulation (63). Interestingly, in a murine sepsis model, while dexamethasone treatment led to 100% survival, rapamycin treatment led to ~40% survival, and combined treatment led to ~50% survival, providing *in vivo* evidence that the pro-inflammatory impact of mTOR inhibition by rapamycin supersedes the anti-inflammatory impact of dexamethasone stimulation of the glucocorticoid receptor (63). With respect to cytokine production, there is a fair amount of consistency regarding the role of mTOR-dependent promotion of IL-10 production as an important brake on the inflammatory cytokine output by DCs.

### Antigen Presentation and T Cell Stimulation

An important prerequisite for a DC's ability to stimulate T cells *in vivo* is its capacity to traffic to secondary lymphoid organs upon activation. While one study has shown that rapamycin limits DC lymph node trafficking in a mouse psoriasis model (64), other studies have shown no impact of rapamycin treatment on CCR7 expression or *in vivo* migratory capacity (47). In general, further studies are needed to better define the role of mTOR in regulating DC chemotaxis and migration, particularly given the high metabolic demand that these processes likely require. With regard to mTOR's impact on DC T cell stimulatory capacity, the published literature indicates that this phenotype is highly dependent on the DC subset in question. One study reported that rapamycin treatment enhanced the ability of TLR7-stimulated human pDCs to promote both the proliferation of CD4+ T cells and the induction of T regulatory cells (48), while other studies support the idea that rapamycin treatment globally suppresses DC capacity to stimulate T lymphocytes (49, 53, 58, 65). A more nuanced look at mTOR's role in T lymphocyte activation has shown an important role for mTOR-mediated Th1/Th2 skewing of CD4+ cells, with the majority of studies demonstrating that mTOR preferentially supports Th2 lymphocyte activation, presumably through its function in promoting IL-10 production by DCs (62, 66, 67). Additionally, mTOR inhibition by rapamycin has been shown to promote T regulatory cell induction both *in vitro* and *in vivo* through DC-dependent action (68).

In contrast to the studies above, we and other have shown that mTOR inhibition can enhance T cell stimulatory capacity in certain contexts (11, 15, 50, 69). mTORC2 deficiency has been documented to augment CD8+ lymphocyte -mediated graft rejection in mice (70), while mice given autologous DCs simulated with LPS in the presences of rapamycin led to a negative impact on T lymphocyte activation and improved graft vs. host survival (71). In GM-CSF -differentiated BMDCs, both mTOR inhibitors enhance LPS-driven DC activation and T cell stimulatory capacity, at least in part through attenuation of mTOR-dependent nitric oxide generation (11, 15). In multiple mouse models of autologous DC vaccination, rapamycin conditioning of DCs enhanced vaccine efficacy to both melanoma tumor challenge (11) and tuberculosis infection (69). This phenotype is not restricted to mouse cells as freshly isolated CD1c+ human myeloid DCs have also been shown to exhibit enhanced T cell proliferation with rapamycin conditioning (50). Nevertheless, whether or not rapamycin treatment can enhance DC autologous vaccination regimens in humans remains to be determined.

### mTOR Control of Mouse iNOS Expression and NO Production

We have previously reviewed the profound impact that DC iNOS expression and NO production has on the metabolism, survival, and immune function of these cells (32). TLR activation induces iNOS expression in mouse BMDCs, and the long-term metabolic commitment to glycolysis in these cells is driven by reversible NO-mediated inhibition of mitochondrial respiration in these cells (12, 15). Furthermore, iNOS inhibition or deletion leads to prolonged post-activation survival and enhanced immunostimulatory capacity in mouse BMDCs (11, 12, 15). Interestingly, several recent studies have shown that mTOR promotion or *Leishmania* infection can downregulate iNOS expression in macrophages *in vivo*, suggesting that regulation of iNOS expression may depend critically on complex factors *in situ* (72, 73). A recent study, investigating the role of innate immune receptor signal strength in modulating DC metabolism and function, showed that stronger stimuli induce higher iNOS expression, NO production, and more dramatic inhibition of mitochondrial respiration (21). In these studies, even though early activation is associated with mTORC1 activity, only strong inflammatory stimuli induce sustained mTORC1 and mTORC2 activation (21). Other studies have suggested that mTOR-driven glycolysis regulates iNOS expression itself (30). These studies showed that both glucose depletion and rapamycin inhibition led to decreased HIF1α and iNOS expression, and promotion of HIF1α activity induced *Nos2* (iNOS) mRNA expression (30). Interestingly, a reciprocal relationship between iNOS and HIF1α was observed as iNOS inhibition or deletion also led to diminished HIF1α expression (30). These studies showed that the relationship between mTOR -mediated metabolic changes and iNOS activity is complicated, but defined an anti-inflammatory effect of glucose metabolism on DC immune function that is dependent on an mTOR/HIF1α/iNOS signaling circuit (30). Furthermore, there is evidence to suggest that T cell depletion of local glucose levels in the tissue microenvironment can impact mTOR/HIF1α/iNOS activity (30). While the contribution of mTOR-mediated iNOS expression and function to DC metabolism is striking, it is noteworthy that even in DC subsets that do not produce NO, mTOR inhibition can augment DC immune function (11, 15, 50).

It noteworthy to consider that only specific subsets of DC populations express iNOS in both mice and humans. GM-CSF -differentiated mouse BMDCs classically induce iNOS expression when stimulated by LPS and IFN-γ (74). From an *in vivo* perspective, monocyte-derived inflammatory DCs (originally termed TNF-a/iNOS-producing-DCs, or “TipDCs”) are potent NO producers and are required to control a number of different types of both bacterial and viral infections (75–77). However, conventional tissue-resident DC subsets in secondary lymphoid organs rarely express iNOS in mice (12, 78, 79), and GM-CSF/IL-4—cultured monocyte-derived human DCs (moDCs) also do not express iNOS (12, 80). Despite these differences, it is clear that human DC populations can express iNOS *in vivo* including blood circulating CD1a^+^ DCs (81) and DCs found in psoriatic skin lesions (82–84). Given the heterogeneity of iNOS expression in DC subsets discussed above, it is clearly important to consider the subsets of DCs under investigation when interpreting the literature and this heterogeneity alone may explain key discrepancies among various studies. Nevertheless, even as a relatively rare subset, iNOS-expressing DCs have important immunological and metabolic consequences in the inflammatory tissue microenvironment that is important to consider (32).

## mTOR Regulation of DC Lifespan and Survival

While mTOR -dependent NO production has been strongly implicated in promoting DC cell death in iNOS-expressing DC subsets (11, 15), there is significant evidence to suggest that mTOR also plays a role in promoting DC survival in other contexts. Treatment of C57BL/6 splenic CD11c+ mature dendritic cells with AMPK activators, which directly antagonize mTOR activity, leads to increased pro-apoptotic molecule expression (85). In addition, rapamycin treatment of splenic CD11c+ mature dendritic cells increased apoptosis, indicating that mTOR promotes cell survival in this system (85). Interestingly, CCR7 expression induced by DC activation leads to inhibitory phosphorylation of AMPK and subsequent activation of mTOR signaling, which supports the post-activation survival of these cells (85). In further support of mTOR serving as a survival promoter in DCs, rapamycin-treated CD34+ hematopoietic progenitor cells resulted in impaired interstitial DC development, indicating that PI3K/mTOR regulates proliferation and survival (86). In addition, human moDCs treated with rapamycin induces decreased expression of anti-apoptotic protein mcl-1 and drives moDC apoptosis (87).

## mTOR Controls Autophagy in DCs

The process of autophagy, whereby cytoplasmic components are ingested, degraded, and their molecular components recycled, plays important roles in DC antigen presentation [recently reviewed in (24)]. It has been elegantly shown that autophagy is a constitutive process in DCs that actively contributes endogenous peptide antigens to MHC-II complexes in the resting state (88). Upon TLR stimulation, the increase in mTOR activity inhibits the formation of the autophagy initiation complex, thereby restricting autophagy rates from the basal state (89). Experts in the field have argued that mTOR-dependent autophagy inhibition leads to a decreased emphasis on endogenous antigen presentation and increased presentation of exogenous antigen (22, 89). In support of this model, IL-4 -mediated induction of autophagy has been shown to augment endogenous antigen presentation on MHC-II molecules (90). Interestingly, while mTOR attenuates autophagy-dependent contributions to antigen presentation, it concomitantly promotes the presentation of exogenously acquired antigens by supporting lysosome acidification and endolysosomal trafficking of MHC-II/peptide complexes to the cell surface (91–93). These findings have clear clinical significance as kidney transplant patients on mTOR-inhibitor therapy exhibit higher levels of alloreactive T cells, possibly due to enhanced autophagy-dependent presentation of donor-endogenous antigen (94). While it seems clear that activated DCs downregulate autophagy in an mTOR -dependent manner, the contribution of autophagy to the nutrient compartment of resting DCs and its counter-regulation by AMPK remains an underexplored topic.

## Concluding Remarks

**Figure 1:**
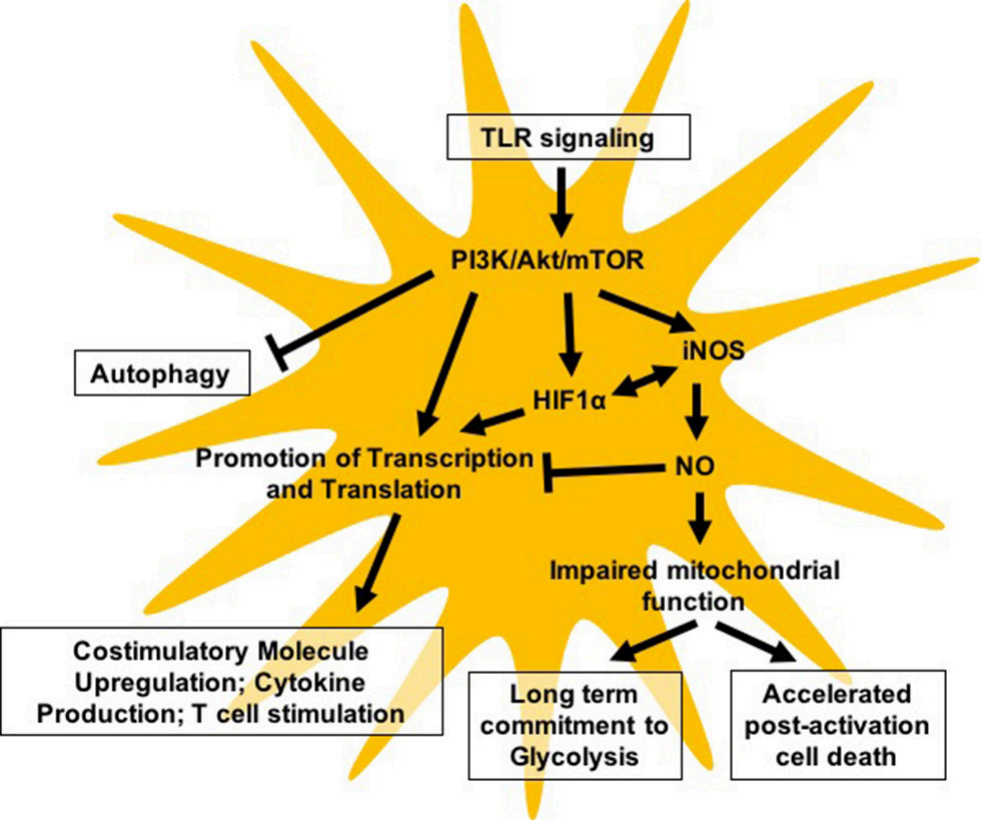
Model highlighting major pathways reported to be regulated either directly or indirectly by mTOR in DC biology.

Given the importance of metabolic changes in supporting the immune activation of DCs, it is not surprising that the central metabolic regulator mTOR plays a critical role in coordinating activation-associated changes in DC metabolism and function (Figure 1). While mTOR has well-documented impacts on DC development, immune effector function, and survival, the challenge in the field rests in understanding the complex and nuanced role that mTOR plays in distinct DC subsets and specific immunological contexts. To this point, it is evident that mTOR can influence DC biology in either a pro-inflammatory or anti-inflammatory direction, which can complicate the interpretation of data where global inhibition of mTOR is employed. Significant aspects of mTOR-mediated regulation of DC biology that would benefit from further investigation include the role of mTOR in nutrient flux in both basal and activated conditions, the cross-regulation of these processes by AMPK, the contribution of mTOR signaling to lipid metabolism, and a further delineation of differential mTOR signaling in distinct DC subsets in both mouse and human cells. We look forward with interest to the ongoing work in the field that will help resolve some of these discrepancies and better clarify the distinct contribution of mTOR signaling to the heterogeneous family of DCs in the mammalian immune system.

## Author Contributions

All authors listed have made a substantial, direct and intellectual contribution to the work, and approved it for publication.

### Conflict of Interest Statement

The authors declare that the research was conducted in the absence of any commercial or financial relationships that could be construed as a potential conflict of interest.
